# Treated Seawater as a Magnesium Source for Phosphorous Recovery from Wastewater—A Feasibility and Cost Analysis

**DOI:** 10.3390/membranes6040054

**Published:** 2016-12-13

**Authors:** Cejna Anna Quist-Jensen, Mads Koustrup Jørgensen, Morten Lykkegaard Christensen

**Affiliations:** Department of Chemistry and Bioscience—Section for Chemistry, Aalborg University, Fredrik Bajers vej 7H, Aalborg Ø 9220, Denmark; mkj@bio.aau.dk (M.K.J.); mlc@bio.aau.dk (M.L.C.)

**Keywords:** resource recovery, integrated membrane systems, nanofiltration, membrane distillation, membrane crystallization

## Abstract

Conventional resources of phosphorous are at high risk of depletion in the near future due to current practices of its exploitation, thus new and improved exploration methodologies need to be developed to ensure phosphorous security. Today, some treatment plants recover phosphorous from municipal wastewater as struvite (MgNH_4_PO_4_·6H_2_O). Magnesium is often added to the wastewater as MgCl_2_·6H_2_O to facilitate the phosphorous recovery. However, the use of magnesium increases the costs of the process and is not aligned with sustainable development, therefore, alternative magnesium sources have to be found. The current study analyzes the feasibility of integrated membrane processes for magnesium recovery from seawater for utilization in the phosphorous recovery process. The integrated membrane systems consist of nanofiltration (NF), membrane distillation (MD), and membrane crystallization (MCr). The lowest associated cost is found for standalone NF treatment. However, the additional treatment with MD and MCr produces fresh water and salts like NaCl or potentially other valuable minerals at the expense of low-grade heat.

## 1. Introduction

Transition towards circular economy is a high-profile action planned worldwide. In particular, the redesign of conventional processes and practices to turn waste into resources is highlighted due to the impending risk of energy, water, and minerals scarcity. The objective is to increase and improve sustainable recovery, and use and recycling of methods to reduce future threats of depletion. Wastewater treatment can, if the recycling of nutrients is enhanced, help to reduce conventional mining. For instance, phosphorous resources are at high risk of depletion [1], thus novel methodologies have to be developed to ensure phosphorous security. In order to meet standards of phosphorous concentration in effluent, some wastewater treatment plants recover phosphorous as struvite (MgNH_4_PO_4_·6H_2_O), due to the advantage of being a slow-release fertilizer [2,3]. Some interesting methodologies to enhance the precipitation of struvite include the addition of magnesium by means of fluidized bed reactors at an industrial scale in many countries including Denmark, Japan (PHOSNIX), Canada (Ostara), Netherland (PHOSPAQ™, AirPrex), and Germany (Seaborne, AirPrex) [4]. Today, the recovered struvite is sold as commercialized fertilizers. Magnesium is often added as MgCl_2_·6H_2_O or other magnesium salts, which, nevertheless, stresses the overall sustainability and economic feasibility of struvite recovery. In order to make the entire phosphorous recovery process fully aligned with sustainable development, alternative methods have to be developed. Several studies have addressed alternative and cost-effective magnesium sources for phosphorous (P) removal [4]. Some studies suggest bittern; the byproduct from salt manufacturing [5,6], wood ash [7], or seawater and brine [8,9]. However, the use of different magnesium sources will depend on the applied post-treatment of the wastewater and the content of heavy metals [7] or other unintended ions in the source. For instance, seawater is a complex solution containing several other ions including chloride, which creates problems for the downstream wastewater treatment such as the anaerobic ammonium oxidation (anammox) process or flocculation process caused by high ionic strength. Previously, it has been shown that high chloride concentrations in the anammox process inhibit the nitrogen removal capability [10]. Consequently, seawater treatment is required before addition to the wastewater.

The objective of the current study is to analyze the economic feasibility of treating seawater to recover magnesium to be used in the phosphorous recovery process from wastewater. The focus is to increase magnesium concentration in seawater, while avoiding high chloride concentration. Membrane operations, such as nanofiltration (NF), can be a potential process in this regard as it allows separation of magnesium (Mg) and chloride (Cl) ions, depending on the properties of the utilized membrane. Moreover, NF is operated at lower applied pressure compared to reverse osmosis (RO), thus it is also feasible from energy and economic perspectives. A similar approach has previously been highlighted in the study of Telzhensky et al. [11] and Lahav et al. [12]. The current study extends previous research to also include integrated membrane systems consisting of NF, membrane distillation (MD), and membrane crystallization (MCr). Unlike RO and NF, MD and MCr are not denoted as pressure-driven membrane operations. Instead, they are operated by using low-grade heat, which reduces the energy consumption. MD and MCr are based on a temperature gradient across a hydrophobic microporous membrane. Due to the hydrophobic nature of the membrane, only vapor can go through the membrane. MD and MCr have several advantages with respect to traditional distillation and crystallization processes, for example, they can be operated at low temperatures thus waste heat can be utilized; moreover, MD and MCr produce a fresh water stream that does not require additional treatment before use [13]. The difference between MD and MCr is that MCr operates at higher recovery factors, thus crystals may nucleate and grow in solution. In this study, MD serves to increase the concentration of magnesium ions and act as a volume reduction application, so the wastewater is less diluted with respect to standalone nanofiltration. Eventually, if MD is continued, NaCl can be crystallized and removed from seawater—at this stage the process is denoted as MCr instead of MD. Therefore, the MCr process allows higher Mg and Cl separation with respect to MD and NF.

## 2. Materials and Methods

The objective of this study is to evaluate the potential of seawater treatment with specific focus on magnesium ions. The evaluation is based on the quantities in which magnesium has to be added to wastewater in order to replace current practices of using a struvite recovery unit located at a Danish wastewater treatment plant. The required quantity of reject water (Table 1) to be treated might be between 200–1000 m^3^/day, depending on the plant. In the current study, a flow of 500 m^3^ reject water/day has been considered. The composition of reject water (Table 1) applies to the regional differences and upstream treatment, thus averaged numbers and simplifications have been considered in the current study. The intake of seawater has been based upon the volume of reject water and that the magnesium to phosphate ratio should be 1:1.3, as used for struvite recovery at Danish wastewater treatment plants. Seawater composition (Table 2) has been estimated as 20 g/L of dissolved solids, equal to seawater near the Danish coast. 

**Figure 1:**
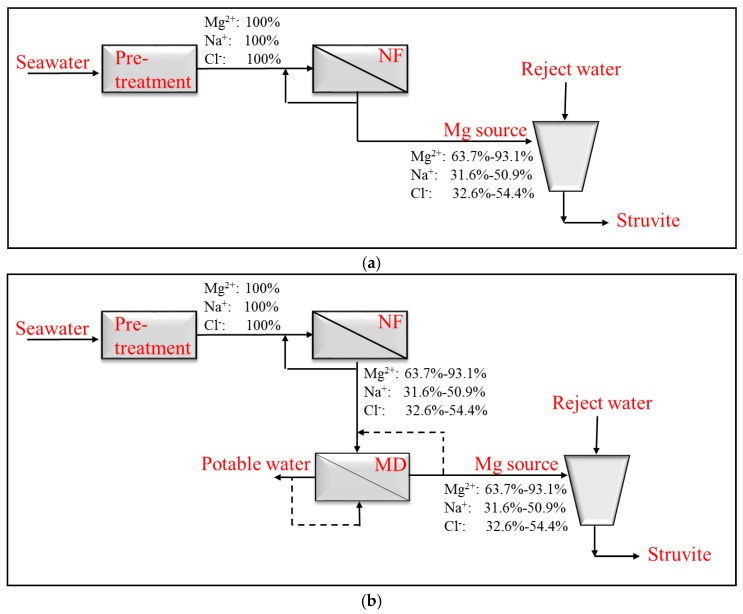

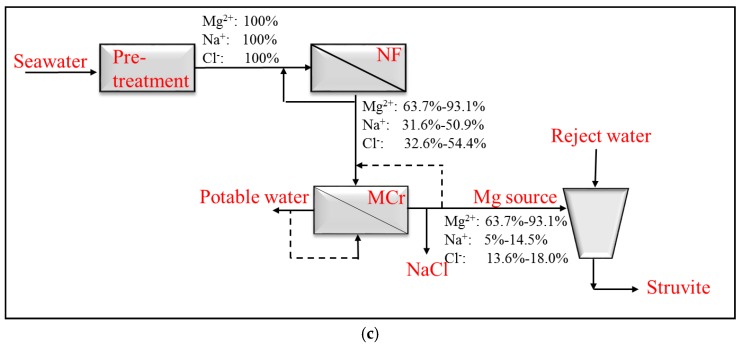
(**a**) Flowsheet 1 (FS1); (**b**) flowsheet 2 (FS2); (**c**) flowsheet 3 (FS3). The flow of ions mentioned in the figure depends on the membrane used and the concentration factor achieved.

This study suggests the use of membrane technology to concentrate seawater and separate magnesium ions from chloride. Three flowsheets (FS) consisting of NF, MD, and MCr have been proposed. FS1 consists of a pretreatment followed by an NF step to separate bivalent and monovalent ions (Figure 1a). FS2 concentrates the NF retentate before adding it to the reject water (Figure 1b), and FS3 concentrates the NF retentate and removes NaCl by introducing MCr (Figure 1c).

The economic evaluation considers only the cost of seawater treatment. The plant used to recover struvite from reject water is assumed to be already established and therefore, buildings and the like have not been taken into account. Three NF membranes with different permeability and retention have been evaluated for FS1, FS2, and FS3 (Table 3 and Table 4). The MD and MCr systems are considered using polypropylene membranes with an average flux of 5 L/(m^2^·h).

The economic evaluation (Equation (1)) considers direct capital costs (auxiliaries, seawater intake and pretreatment system, pumps, membrane costs, heat exchanger cost), indirect capital costs (administration, insurances, field supervision, etc.)—which are estimated as 10% of direct capital costs— and operation and maintenance costs (electricity, labor, membrane replacement, spare costs, chemicals, and steam costs). A detailed description of the economic evaluation including equations and assumptions can be found in Appendix A,

(1) TC=DC+IC+O&M=DC+0.1DC+O&M

where *TC* is the total treatment cost, *DC* and *IC* is the direct and indirect capital cost, respectively, and *O&M* is the operation and maintenance cost.

## 3. Results and Discussion

### 3.1. Dependency of Seawater Intake

**Figure 2:**
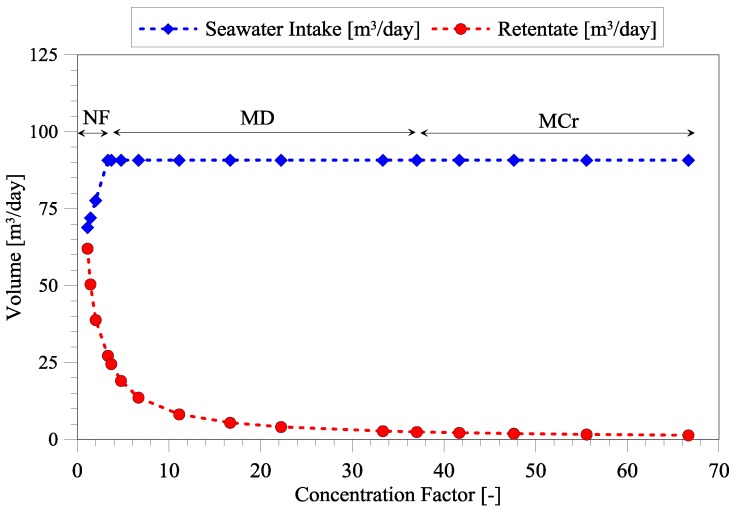
Seawater intake and retentate volume at increasing concentration factor.

The feasibility of treating seawater to obtain a sustainable magnesium source in the phosphorus recovery process has been based upon the treatment of 500 m^3^/day of reject water. Moreover, it is required that after seawater treatment, a P:Mg ratio of 1:1.3 has been achieved. Therefore, the seawater intake has been changed according to these requirements. At first, only the NF membrane from Alfa Laval has been evaluated. The required volume of seawater treated through integrated membrane operations are shown in Figure 2 at an increasing concentration factor (CF) (Equation (2)).

(2) $$CF=\frac{V_{in}}{V_{ret}}$$

where *V_in_* and *V_ret_* are the intake volume and retentate volume, respectively. 

The initial increase in required seawater intake is due to the retention of magnesium ions of the Alfa Laval membrane, which is 85.5% (Table 4), thus some magnesium ions are transferred to the permeate. For this reason, the seawater intake increases from around 70 m^3^/day (at CF = 1) to 90 m^3^/day (at CF = 3.3) in order to maintain the proper P:Mg ratio. In order to maintain the permeability of around 5 L/(m^2^·h·bar) at pressure of ~15 bar, seawater treatment through NF has been stopped at CF of 3.3, equal to permeate removal of 70%. If the concentration factor should be further increased through NF, the energy requirement will also increase. Instead, the NF retentate is treated through MD, denoted as FS2 (Figure 1b). The ion retention of the MD and MCr membrane is theoretically 100%, thus the seawater intake is constant at increasing CF. FS3 is essentially the same as FS2, the only difference is that around a CF of 34, seawater has been concentrated to the saturation point of NaCl and, therefore, the MD process is denoted as MCr.

### 3.2. Magnesium and Chloride Separation

**Figure 3:**
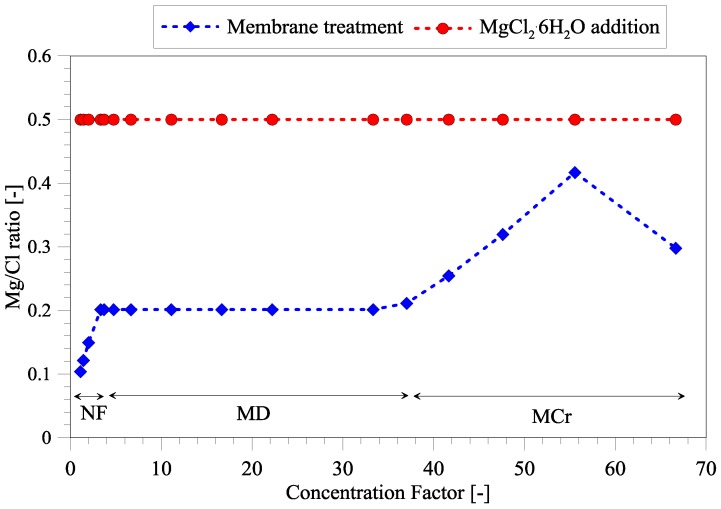
Magnesium and chloride separation at increasing concentration factor.

The volume of retentate decreases as CF increases, thus it can increase the subsequent P recovery, due to less dilution of wastewater. However, the main advantage of seawater treatment is the separation of Mg and Cl ions, which is illustrated in Figure 3. The separation of Mg and Cl ions have been compared to the Mg/Cl ratio when magnesium is added to wastewater in the form of MgCl_2_·6H_2_O. The Mg/Cl ratio in untreated seawater is only 0.1, which is the reason why seawater cannot be added to wastewater without any treatment. The Cl ions affect the downstream processing of wastewater, for instance, in the anammox process. NF treatment is able to increase the Mg/Cl ratio to 0.2. If the operators of the wastewater treatment plant consider this ratio appropriate, the treatment can eventually be stopped (FS1). If a higher Mg/Cl ratio is needed, further treatment can be performed through FS2 or FS3. MD process does not allow separating Mg and Cl, thus the ratio is constant at 0.2 until precipitation of NaCl.

Although NaCl precipitation seems to first occur at a very high CF, this has been extensively proved possible at a lab-scale in the last 10 years [19,20,21] and is closely aligned with the research and perspectives of zero-liquid discharge in the desalination industry. When CF reaches levels above 55, the Mg/Cl ratio starts to decrease. The reason is that magnesium in the form of MgSO_4_·7H_2_O (epsomite) begins to precipitate, as also observed in the studies by Drioli et al. [19] and Macedonio et al. [20]. Depending on how the post-processing stages of the wastewater treatment respond to sulfate ions, it can also be considered to recover magnesium in solid form. However, this is out of the scope of the current paper, which will only consider NaCl removal via MCr technology. The maximum Mg/Cl ratio that FS3 can obtain is around 0.4, which is still slightly lower than the 0.5 achieved through MgCl_2_·6H_2_O addition. In case higher Mg/Cl ratios are required, the process can eventually be integrated with other processes such as electrodialysis. However, this will further increase treatment costs and has not been considered in the present study.

### 3.3. Cost Analysis of Integrated Membrane Systems

**Figure 4:**
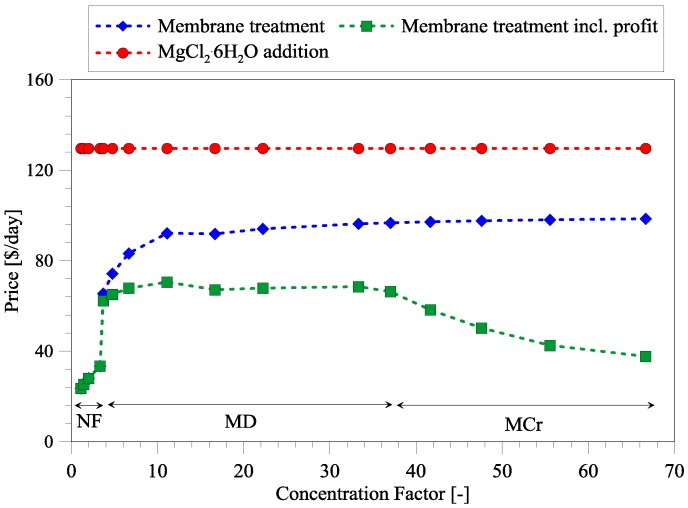
Treatment cost of integrated membrane systems per day compared to the conventional addition of magnesium salt. Membrane treatment including profit is based on selling fresh water produced by the membrane distillation (MD) process and selling of fresh water and NaCl salt produced during membrane crystallization (MCr) process.

The cost of seawater treatment has been estimated according to the procedure described in Appendix A and has been compared to the price of MgCl_2_·6H_2_O addition (Figure 4). The main reason for considering Mg sources other than the conventional addition of MgCl_2_·6H_2_O is the high associated cost (~310 $/ton). In order to treat 500 m^3^/day wastewater, an expenditure of around 130 $/day is required. Therefore, it is interesting to determine if treated seawater can be a competitive Mg source. As shown in Figure 4, the NF treatment is very competitive with respect to MgCl_2_·6H_2_O dosage. The major cost breakdown of NF systems is associated with the electricity required to operate high-pressure pumps. NF is a widely used process in industry and, therefore, a large number of appropriate membranes are available at reasonable prices. The cost of NF treatment ranges from 24 $/day to 34 $/day, depending on the CF, which is also related to seawater intake (Figure 2). If NF is sufficient to separate Mg and Cl ions (Mg/Cl ratio = 0.2), FS1 presents the most feasible with cost reduction of 74% with respect to MgCl_2_·6H_2_O addition. On the other hand, if the required Mg and Cl ratio is above 0.2, FS2 and FS3 should be considered. In Figure 4, it is illustrated that MD and MCr increase the cost significantly. This is mainly due heat energy requirements of MD and MCr processes and due to a higher cost of MD/MCr membranes with respect to NF membranes. To date, no commercially available membranes have been developed specifically for MD/MCr process, thus, commercially available polypropylene membranes have been considered in the current study. However, extensive research is underway to develop and fabricate MD membranes, which can increase the performance and decrease the cost in future. In this study, heating of seawater to 60 °C using steam has been considered, which accounts for the largest share in the cost breakdown of the MD/MCr processes. However, it has to be noted that MD and MCr can be operated using waste heat, which can be available at wastewater treatment plants. In fact, many wastewater treatment plants are currently producing electricity, biogas, and are generating heat from many other processes, which can be a valuable source to reduce the cost of MD and MCr. Heating through steam and the associated cost thereof have been considered, and the treatment cost is still below that associated with the addition of MgCl_2_·6H_2_O.

Furthermore, MD and MCr produce a highly pure fresh water stream, which can be used in other processes, such as cleaning, or can be sold. Moreover, MCr produces high-quality NaCl crystals. The profit of selling water and NaCl has been withdrawn from the treatment cost in Figure 4 (green curve). The exact numbers used to calculate the profit (i.e., price of water and NaCl) can be found in Appendix A, which is, of course, dependent upon regional differences. The profit reduces the treatment cost of MD and MCr, but cannot meet the cost of only NF treatment. In comparison, precipitation of magnesium from seawater (Mg(OH)_2_) by lime or other reagents can be an alternative method. However, Alamdari et al. stated some drawbacks of using lime as precipitation agent, such as low purity and precipitation of calcium sulfate if the water contains a large amount of sulfate ions [22]. Therefore, other and more expensive precipitation agents or additional treatment are required to obtain a suitable magnesium product. The advantage of using Mg(OH)_2_) in the phosphorous recovery process is that it can help in adjusting pH. Nevertheless, pH and magnesium addition cannot be controlled independently, and Mg(OH)_2_ has a slower dissociation with respect to MgCl_2_·6H_2_O, which then requires smaller particle sizes and more mixing [23,24]. Therefore, recovery of magnesium from seawater might need additional steps to convert Mg(OH)_2_ into other magnesium compounds, which will then increase the production cost further. Therefore, this study has solely focused on minimizing the use of chemicals by means of membrane-based magnesium recovery and compared these results to the commonly used procedure of adding MgCl_2_·6H_2_O.

### 3.4. Efficiency of Phosphorous Removal Using Treated Seawater as Mg Source

**Figure 5:**
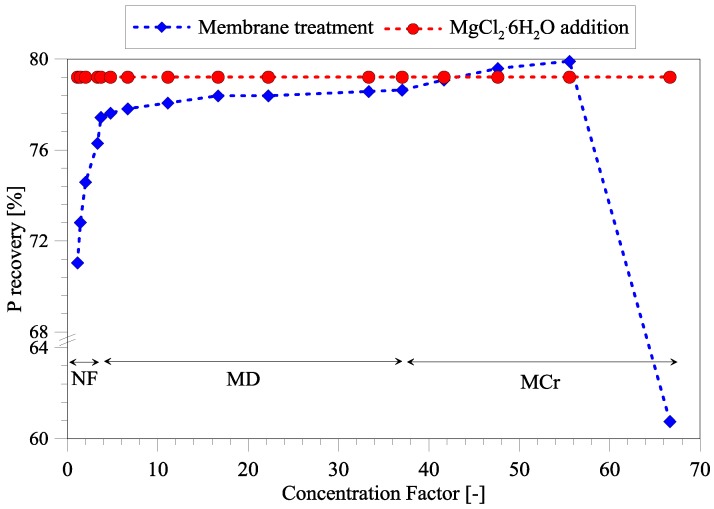
The theoretical phosphorous recovery percentage through addition of treated seawater to reject water. The phosphorous recovery has been compared to the conventional addition of magnesium salts.

Beside cost considerations, the conclusive decision on the extent of treatment depends on the required Mg/Cl ratio and the amount of phosphorous (P) recovery. P recovery from wastewater has been simulated through PHREEQC [25], taking into account the composition and volume of treated seawater and wastewater. The difference between addition of MgCl_2_·6H_2_O and treated seawater are illustrated in Figure 5. P recovery through addition of MgCl_2_·6H_2_O approaches a theoretical value of ~79%. As shown, NF treatment increases P recovery from ~71% to ~77.5%, which is slightly lower than what can be achieved through addition of MgCl_2_·6H_2_O. MD treatment increases P recovery only slightly due to volume reduction. Only MCr treatment is able to meet the same P recovery as MgCl_2_·6H_2_O addition. However, a drastic drop in P recovery is observed when CF is above 55 due to the precipitation of magnesium salts, which have then been removed from the treated seawater and hence not added to the reject water.

Operators of wastewater treatment plants can decide whether NF-treated seawater is enough to meet the requirements of P recovery in the effluent or if additional treatment through MD/MCr is required. Moreover, the practical comparison of treated seawater and addition of MgCl_2_·6H_2_O has to be accomplished.

### 3.5. Effect of Initial Seawater Composition

**Figure 6:**
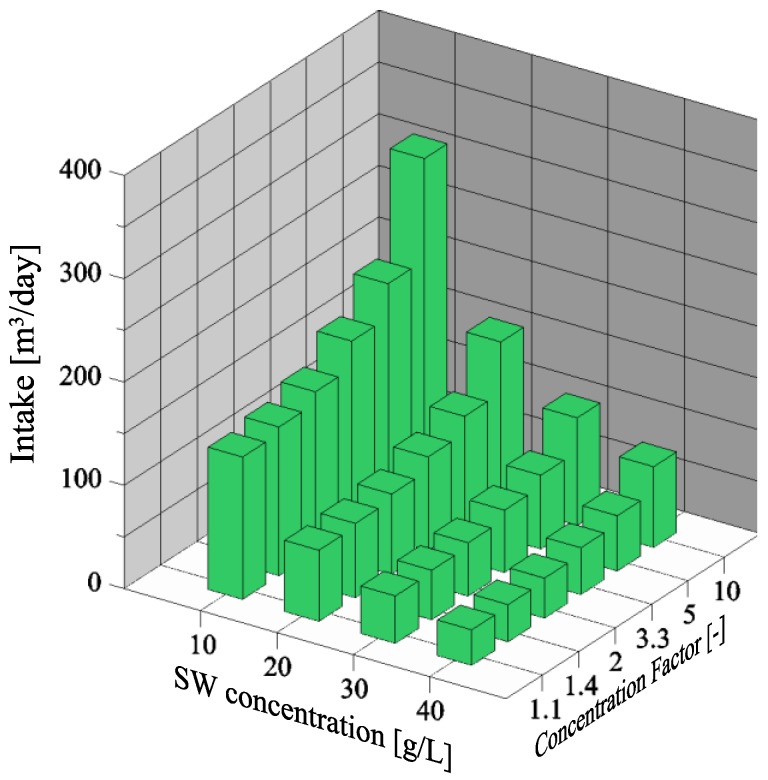
Seawater (SW) intake at different initial seawater concentrations and at increasing concentration factors.

**Figure 7:**
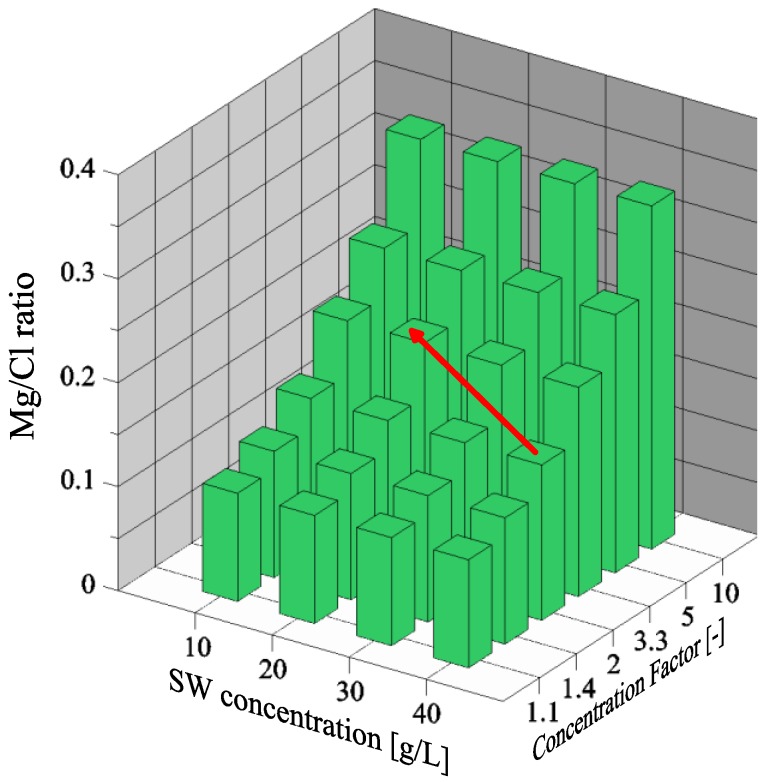
Magnesium and chloride separation at different initial seawater concentrations and at increasing concentration factors. The red arrow symbolizes the possibility to increase the Mg/Cl ratio by using lower concentrations of seawater.

**Figure 8:**
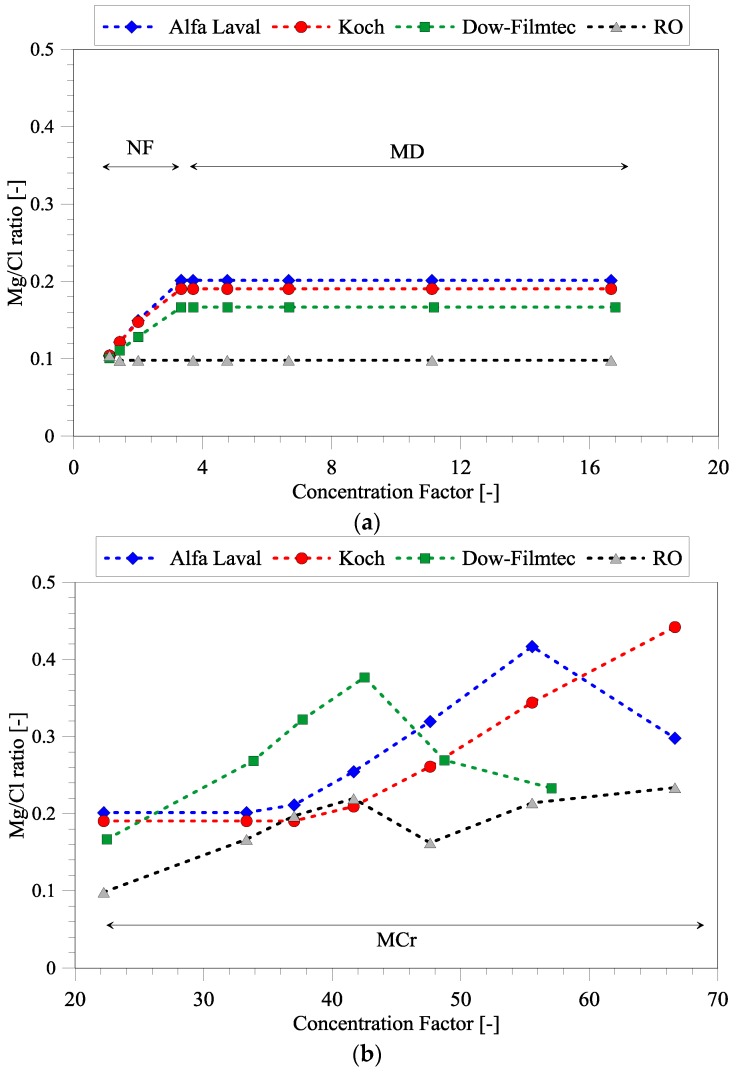
Evaluation of different NF and reverse osmosis (RO) membranes in terms of their separation of Mg and Cl. (**a**) Concentration factor (CF) from 0 to 20, which is the FS1 and FS2 and (**b**) CF from 20 to 70, which is related to FS3.

Another factor effecting the efficiency and treatment of seawater is the initial concentration of salts present in seawater. Change in concentration, ranging from 10 g/L to 40 g/L, have been evaluated (only for NF treatment), where the ratio of each ion has been kept constant as shown in Table 2. As illustrated in Figure 6, the initial concentration impacts the intake of seawater, thus higher concentration minimizes the volume of intake. On the contrary, the Mg/Cl ratio is not affected directly by increasing the initial concentration. However, higher concentration might reduce the highest achievable concentration factor (this cannot be seen directly from Figure 6 or Figure 7), due to higher applied pressure in NF operation. Therefore, it might be possible to achieve a CF of 3.3 with initial seawater concentration of 20 g/L instead of a CF of only 2.0 at a concentration of 40 g/L (displayed as the red arrow in Figure 8).

### 3.6. Comparison of Different NF Membranes

The studies (described above) on the feasibility of seawater treatment have been carried out by using NF membranes from Alfa Laval with ion-retention given in Table 4. Nevertheless, the use of other membranes can change the optimal treatment options, accordingly two NF membranes from Dow-Filmtec and Koch Membrane Systems and an RO membrane (ion retention above 98.9% [26]) have also been briefly evaluated. The Mg/Cl ratio can be controlled by changing the NF membrane as shown in Figure 8. The Dow-Filmtec membrane has the lowest separation of Mg and Cl (Figure 8a) compared to the other NF membranes, but this allows precipitation of NaCl at lower CF (Figure 8b). Therefore, this membrane can be considered if the Mg/Cl ratio should be higher than what NF treatment (FS1) can achieve, but still at lower CFs compared to the other NF membranes. Another possibility is to use an RO membrane instead of NF. RO can normally only reach CFs of around 2, the value utilized in this study. Standalone RO cannot separate Mg and Cl (Figure 8a), but might allow more separation of NaCl during MCr treatment. However, according to Figure 8b, this is not the case. In fact, the use of RO seems not to be the best option, as the magnesium salts (brucite and epsomite) are also precipitating together with NaCl. Nevertheless, brucite crystallization might be inhibited by pH control or can be recovered and added directly to reject water, thus increasing the Mg/Cl ratio more than what is shown in Figure 8b.

## 4. Conclusions

This study shows that it is economically feasible to treat seawater to obtain a highly concentrated magnesium source that can be added to wastewater for phosphorous recovery. The cost of NF treatment of seawater is in the range of 0.10 $/kg struvite, MD around 0.22 $/kg struvite (including profit) and MCr as low as 0.15 $/kg struvite (including profit). In comparison to addition of MgCl_2_·6H_2_O, which is around 0.42 $/kg struvite, the overall treatment cost through integrated membrane systems is more feasible. The cost of MD and MCr treatment is higher than NF, but MD and MCr obtain higher Mg/Cl ratios. Moreover, MD and MCr treatment can also reduce the volume of seawater brine which can be beneficial from environmental point of view. The cost of MD and MCr processes can eventually be significantly reduced by utilizing low-grade heat available at the wastewater treatment plants.

## Figures and Tables

**Table 1:** Properties of typical reject water.

Component	Range in Reject Water	Used in This Study
NH_4_^+^ (mg/L)	120–2043 [14,15,16]	900
PO_4_^3−^ (mg/L)	15–484 [14,16]	300
Ca^2+^ (mg/L)	<50	–
Na^+^ (mg/L)	<200	–
K^+^ (mg/L)	<200	–
Mg^2+^ (mg/L)	<30	–
Suspended matter (sludge, polymer) (mg/L)	100–200	–
Required Mg/P ratio	1.3	1.3
pH (controlled by NaOH)	7.5	7.5

**Table 2:** Seawater composition.

Component	Composition (%)	Composition at 20 mg/L Dissolved Solids (mg/L)
Chlorine Cl^−^	55.03	11,006
Potassium K^+^	1.11	222
Magnesium Mg^2+^	3.68	736
Sodium Na^+^	30.59	6118
Sulfate SO_4_^2−^	7.68	1536
Calcium Ca^2+^	1.18	236
Bicarbonate HCO_3_^−^	0.41	82

**Table 3:** Permeability of the considered nanofiltration (NF) membranes [17,18].

Membrane Performance	NF99HF (Alfa Laval)	K-SR2 (Koch Membrane Systems)	NF90 (Dow Filmtec)
Permeability (L/(m^2^·h·bar))	5.2	7.3	1.08

**Table 4:** Retention of the considered NF membranes [17,18].

Component	NF99HF (Alfa Laval)	K-SR2 (Koch Membrane Systems)	NF90 (Dow Filmtec)
Cl^−^ (%)	24.4	11.5	64.1
K^+^ (%)	20	11	53
Mg^2+^ (%)	85.5	75.6	96.8
Na^+^ (%)	14.2	7.1	58.7
SO_4_^2−^ (%)	97.3	97	96.7
Ca^2+^ (%)	67.3	59	94.4
HCO_3_^2−^ (%)	57	40	85

## Appendix A

In Table A1, a general description of the considered seawater treatment plant is given together with the assumptions made in order to perform the cost analysis of integrated membrane systems.

**Table A1:** Description and assumptions made to perform cost analysis.

General Plant Description
Plant life time (*n*) = 15 years
Interest rate (*i*) = 0.15
Amortization (*ɑ*) $=\frac{i{\left(1+i\right)}^{n}}{{\left(1+i\right)}^{n}-1}$
Plant availability (*f*) = 0.7
Pressure pretreatment (P_pre_) = 5 × 10^5^ Pa
**NF Plant Description**
Pressure NF (P_NF_) =1.5 × 10^6^ Pa
Pressure drop NF (ΔP_NF_) = 0.5
Pumps efficiencies (η_pump_) = 0.7
Recovery factor (RF_NF_) = 70%
Flux (J_NF_) = 30 L/(m^2^·h)
Area NF module (A_NF_) = 36 m^2^ (8 inch module)
Cost of NF module (C_NF mod_) = 1500 $
NF membrane replacement = 20%
NF maximum concentration factor = 3.3
**MD/MCr Plant Description**
Pressure MD (P_MD_) = 1.5 × 10^5^ Pa
Flux (J_NF_) = 5 L/(m^2^·h)
Cost of MD membrane (C_MD membrane_) = 90 $/m^2^
MD membrane replacement = 35%
Temperature NF retentate (T_NF_) = 15 °C
Temperature MD retentate (T_MD_) = 60 °C
Heat capacity water (C_p_) = 4181.3 J/(kg·K)
Over all heat transfer coefficient (U) = 300 W/(m^2^·K)
Heat exchanger efficiency (η_hex_) = 0.8
Heat exchanger cost (c _hex_) = 2000 $/m^2^
Latent heat of vaporization (λ_vap_) = 2260 kJ/kg
Steam cost (C_steam_) = 0.00705 $/kg
**Cost Assumptions**
Electricity (Danish industry) (c_elec_): 0.1269 $/kWh
Labor (c_labor_): 0.05 $/m^3^
Spare (c_spare_): 0.033 $/m^3^
Chemicals (c_chemicals_): 0.025 $/m3
MgCl_2_·6H_2_O: 310.6 $/ton
Struvite: 379 $/ton
Fresh water (From MD/MCr process): 1.6 $/m^3^
NaCl (From MCr process): 121.3 $/ton

In the section given below, a description on the different costs related to Equation (1) has been highlighted, which includes direct capital cost, indirect capital cost and operations and maintenance costs.

### Appendix A.1. Direct Capital Costs (DC)

#### Appendix A.1.1. Auxiliary Including Pipe Lines, Tanks etc. (C_aux_) [27]

(A1) $$C_{aux}=3608{F_{SW}}^{0.85}+908N_{\mathrm{mod}}\times 1.136$$

*F_sw_* is seawater flow rate in m^3^/h, *N_mod_* is number of NF modules, and 1.136 is to convert € to $.

#### Appendix A.1.2. Intake and Pretreatment

(A2) $$C_{in}=0.15DC$$

(A3) $$C_{pre}=0.2DC$$

#### Appendix A.1.3. Pumps [28]

(A4) $$C_{pump,pre}=4.78\times 10^{-6}\times F_{SW}\times P_{pre}$$

(A5) $$C_{pump,NF}=4.78\times 10^{-6}\times F_{SW}\times P_{NF}$$

(A6) $$C_{pump,MD}=4.78\times 10^{-6}\times F_{MD}\times P_{MD}$$

where *F_MD_* is the inlet flow to the MD module in m^3^/h.

#### Appendix A.1.4. Membrane Costs

(A7) $$C_{tot,NFmembrane}=N_{\mathrm{mod}}\times C_{NF,\mathrm{mod}}$$

(A8) $$C_{tot,MDmembrane}=A_{\mathrm{mod}}\times C_{MD,membrane}$$

where *A_MD_* is the required MD membrane area in m^2^.

The following estimations are only applicable when MD or MCr are introduced (FS2 and FS3) and are related to the heating of the NF retentate (*T_NF_*) to the required temperature in the MD/MCr module (*T_i,MD_*).

(A9) $$Q_{MD}=\frac{F_{MD}\times C_{p}(T_{MD}-T_{NF})\times 1000}{3.6\times 10^{6}\times \eta_{hex}}$$

The heat exchanger area (*A_hex_*) and total cost can be based on the following equations:

(A10) $$A_{hex}=\frac{Q_{MD}\times 1000}{U\times T_{avg}},\text{ where }T_{avg}=\frac{T_{NF}-T_{MD}}{\ln\frac{T_{NF}}{T_{MD}}}$$

(A11) $$C_{tot,hex}=A_{hex}\times c_{hex}$$

Furthermore, 10% has been added to the total DC cost, which considers flexibility in the constructions.

### Appendix A.2. Indirect Capital Costs (IC)

IC includes administration, insurances, field supervision, and others, and has been assumed to be 10% of DC.

### Appendix A.3. Operation and Maintenance (O&M)

#### Appendix A.3.1. Electricity

Pump energy for intake/pretreatment, NF filtration, and MD filtration [29]. No pumping for post-treatment has been considered.

(A12) $$E_{pump,pre}(kW)=\frac{F_{SW}\times P_{pre}}{3.6\times 10^{6}\times \eta_{pump}}$$

(A13) $$E_{pump,NF}(kW)=\frac{F_{SW}\times P_{NF}}{3.6\times 10^{6}\times \eta_{pump}}$$

(A14) $$E_{pump,MD}(kW)=\frac{F_{MD}\times P_{MD}}{3.6\times 10^{6}\times \eta_{pump}}$$

For NF, the feed-and-bleed system has been considered, whereas in MD the feed goes through the membrane module once. Therefore, pumping energy for recirculation for the NF step has also been taken into account. Recirculation energy depends on the pressure drop (*ΔP*) in the module, which has been set to 0.5 × 10^5^ Pa.

(A15) $$E_{pump,recirc}(kW)=\frac{F_{recirc}\times \Delta P_{NF}}{3.6\times 10^{6}\times \eta_{pump}}$$

(A16) $$F_{recirc}=100\times F_{f}$$

(A17) $$E_{tot}(kW)=E_{pump,pre}+E_{pump,NF}+E_{pump,recirc}+E_{pump,MD}$$

(A18) $$C_{pump,tot}(\$/year)=E_{tot}\times c_{elec}\times f\times 24\times 365$$

#### Appendix A.3.2. Labor [26]

(A19) $$C_{labor,tot}(\$/year)=c_{labor}\times F_{SW}\times f\times 24\times 365$$

#### Appendix A.3.3. Membrane Replacement

The NF membrane replacement has been chosen as 20% each year, corresponding to an average lifetime of 5 years. The MD membrane is not yet commercialized, thus the replacement has been chosen as 35% (~3 years lifetime).

(A20) $$C_{replacement}(\$/year)=c_{tot,membranes}\times replacement$$

#### Appendix A.3.4. Spare Costs [28]

(A21) $$C_{spare,tot}(\$/year)=c_{spares}\times F_{SW}\times f\times 24\times 365$$

#### Appendix A.3.5. Chemical Costs

(A22) $$C_{chemicals,tot}(\$/year)=c_{chemicals}\times F_{SW}\times f\times 24\times 365$$

#### Appendix A.3.6. Steam Costs

The steam consumption is given by the following equation [26]

(A23) $$S=\frac{Q_{MD}\times 3600}{\lambda_{vap}}$$

(A24) $$C_{steam,tot}(\$/year)=c_{steam}\times S\times f\times 24\times 365$$
